# Olfactory testing does not predict β-amyloid, MRI measures of neurodegeneration or vascular pathology in the British 1946 birth cohort

**DOI:** 10.1007/s00415-020-10004-4

**Published:** 2020-06-24

**Authors:** Sarah M. Buchanan, Thomas D. Parker, Christopher A. Lane, Ashvini Keshavan, Sarah E. Keuss, Kirsty Lu, Sarah-Naomi James, Heidi Murray-Smith, Andrew Wong, Jennifer Nicholas, David M. Cash, Ian B. Malone, William Coath, David L. Thomas, Carole Sudre, Nick C. Fox, Marcus Richards, Jonathan M. Schott

**Affiliations:** 1 Dementia Research Centre, Department of Neurodegenerative Disease, UCL Queen Square Institute of Neurology, University College London, London, WC1N 3BG UK; 2 MRC Unit for Lifelong Health and Ageing at UCL, London, UK; 3 London School of Hygiene and Tropical Medicine, University of London, London, UK; 4 KCL School of Biomedical Engineering and Imaging Sciences, London, UK

**Keywords:** Alzheimer’s disease, Olfactory impairment, Neuroimaging

## Abstract

**Objective:**

To explore the value of olfactory identification deficits as a predictor of cerebral β-amyloid status and other markers of brain health in cognitively normal adults aged ~ 70 years.

**Methods:**

Cross-sectional observational cohort study. 389 largely healthy and cognitively normal older adults were recruited from the MRC National Survey of Health and Development (1946 British Birth cohort) and investigated for olfactory identification deficits, as measured by the University of Pennsylvania Smell Identification Test. Outcome measures were imaging markers of brain health derived from 3 T MRI scanning (cortical thickness, entorhinal cortex thickness, white matter hyperintensity volumes); ^18^F florbetapir amyloid-PET scanning; and cognitive testing results. Participants were assessed at a single centre between March 2015 and January 2018.

**Results:**

Mean (± SD) age was 70.6 (± 0.7) years, 50.8% were female. 64.5% had hyposmia and 2.6% anosmia. Olfaction showed no association with β-amyloid status, hippocampal volume, entorhinal cortex thickness, AD signature cortical thickness, white matter hyperintensity volume, or cognition.

**Conclusion and relevance:**

In the early 70s, olfactory function is not a reliable predictor of a range of imaging and cognitive measures of preclinical AD. Olfactory identification deficits are not likely to be a useful means of identifying asymptomatic amyloidosis. Further studies are required to assess if change in olfaction may be a proximity marker for the development of cognitive impairment.

**Electronic supplementary material:**

The online version of this article (10.1007/s00415-020-10004-4) contains supplementary material, which is available to authorized users.

## Introduction

Simple, non-invasive markers of preclinical Alzheimer’s disease (AD) are needed. Odour identification (OI) deficits have been proposed as a potential risk marker for AD. Clinically, individuals diagnosed with AD and mild cognitive impairment (MCI) have poorer OI, and OI deficits are associated with cognitive decline and conversion to MCI and AD [1]; and AD pathology affects olfactory pathways in older adults [2] and animal models [3].

While the evidence for these associations in clinically defined groups is strong, the evidence regarding imaging biomarkers is more mixed. Table 1 summarises the previous literature investigating associations between OI and imaging markers of preclinical AD. Considering the two largest cohorts, Vassilaki et al. [4] and Growdon et al. [5] each found associations between poorer OI and imaging markers of neurodegeneration. Amyloid status was positively associated with poorer OI in the former, and at trend level in the latter study. In smaller studies, associations were not found [6, 7], or only seen when individuals with MCI or AD were included in pooled analyses [8, 9]. Associations between poorer OI and AD signature cortical thickness, and lower hippocampal volumes have been described [4, 7, 10, 11]. Associations with entorhinal cortex thickness or white matter hyperintensity volume have been present or absent in various studies [4–6, 10, 11].

**Table 1:** Summary of studies investigating associations between imaging markers of β-amyloid, neurodegeneration or vascular pathology and olfactory testing in non-demented cohorts

Study	Year	Region	Outcome measures	Olfactory test	Design	*n*	Mean age, years (SD, if available)	% female	Key findings
Devanand et al. [10]	2010	USA	sMRI, cognitive tests	UPSIT	Cross-sectional	1092 (802 CN, 120 naMCI, 170 aMCI)	80.5 (5.8)	70	Lower OI associated with lower hippocampal volumes and cognitive test results, but not WMH or entorhinal cortex volumes
Bahar-Fuchs et al. [9]	2010	Australia	Amyloid-PET (PiB)	6 items from the UPSIT	Cross-sectional	63 (19 CN, 24 aMCI, 20 AD)	73.6	57	Higher PiB-PET SUVR associated with lower OI in pooled analyses; however, no relationship found within each cognitive group
Growdon et al. [5]	2015	USA	Amyloid-PET (PiB), sMRI, Cognitive tests (composite)	UPSIT	Cross-sectional	215 (all CN)	73.9 (5.9)	59.1	Poorer OI associated with lower hippocampal and entorhinal cortex volumes, and cognitive scores. Association with amyloid status at trend level
Dhilla Albers et al. [6]	2016	USA	Amyloid-PET (PiB) (*n* = 41), sMRI (*n* = 49) cognitive tests	Two novel olfactory tests	Longitudinal follow-up of cognition	183 (70 CN, 74 SCD, 29 MCI, 10 AD)	76.7	60.8	Poor performance associated with lower hippocampal and entorhinal cortex volumes (pooling all cognitive groups) but not with amyloid status; also associated with longitudinal cognitive decline in CN group
Vassilaki et al. [4]	2017	USA	Amyloid-PET (PiB) (*n* = 306), FDG-PET (*n* = 305), sMRI (*n* = 829)	B-SIT	Cross -sectional	829 (all CN)	79.2	48.5	Anosmia (B-SIT < 6) (but not hyposmia) associated with abnormal PiB-PET and sMRI measures (hippocampal volume, AD signature CT). Continuous B-SIT scores also associated with sMRI measures
Risacher et al. [7]	2017	USA	Amyloid-PET (florbetapir/ florbetaben); Tau-PET (flortaucipir), sMRI	UPSIT	Cross-sectional	34 (19 CN, 10 SCD, 5 MCI)	70.8	64.7	OI not associated with amyloid-PET across sample or any subgroup. Poorer OI associated with tau deposition in the temporal lobe across pooled sample, and in CN plus SCD group. Lower temporal, hippocampal and entorhinal GM volumes across pooled sample
Heinrich et al. [11]	2018	France	sMRI, cognitive tests	B-SIT	Cross-sectional	75 MCI	77.1 (6.2)	74.7	B-SIT < 8 associated with lower hippocampal volume, higher WMH rating, lower MMSE and FCSRT
Kriesl et al. [8]	2018	USA	Amyloid-PET (PiB), Cognitive tests (composite)	UPSIT	Longitudinal follow-up of cognition	71 (25 CN, 46 MCI)	68.5 (7.5)	58	UPSIT < 35 had NPV 100%, PPV 41% for binary amyloid status. UPSIT < 35 predicted memory decline in MCI but not CN participants

*sMRI* structural MRI, *UPSIT* University of Pennsylvania Smell Identification Test, *CN* cognitively normal, *MCI* mild cognitive impairment, *aMCI* amnestic MCI, *naMCI* non-amnestic MCI, *OI* odour identification, *WMH* white matter hyperintensity, *PET* positron emission tomography, *PiB* Pittsburgh compound B tracer, *AD* Alzheimer’s Disease, *CT* cortical thickness, *SUVR* standard uptake value ratio, *FDG* fluorodeoxyglucose, B-*SIT* Brief Smell Identification Test (a validated 12 item selection from the UPSIT), *PPV* positive predictive value, *NPV* negative predictive value, *SCD* subjective cognitive decline, *MMSE* mini-mental status examination, *FCSRT* Free and Cued Selective Reminding Test, *GM* grey matter

A useful marker for preclinical AD would be positive early in the disease course, allowing a window for treatment. As the prevalence of AD pathology increases steeply with age, younger cohorts may be useful to investigate the earlier stages of disease.

In the current study, we explored associations between OI and markers of cerebral β-amyloid deposition (using ^18^F-florbetapir PET scanning), neurodegeneration, and cognition in a uniquely well-characterised cohort of near identical age drawn from the MRC National Survey of Health and Development (NSHD; the British 1946 birth cohort).

## Methods

### Participants

The Insight 46 study included 502 older adults recruited from the NSHD [12], a representative sample of singleton births in one week in March 1946 originally comprising 5326 individuals who have been followed prospectively throughout their lives [13]. Ethical approval was granted by the National Research Ethics Service Committee London (reference 14/LO/1173); participants provided written informed consent.

Participants attended a one-day visit at University College London between May 2016 and January 2018 (age 69–71 years). The cohort profile and recruitment information has been published [14]. We excluded participants without high-quality imaging (T1-weighted MRI and amyloid-PET), and those with mild cognitive impairment (MCI), neurodegenerative conditions, or conditions likely to affect olfactory function including previous sinus surgery or upper respiratory tract infection (Supplementary data).

### Olfactory testing

The University of Pennsylvania Smell Identification Test (UPSIT) is a validated “scratch-and-sniff” test comprising 40 micro-encapsulated odorants, with four-option forced-choice answers [15]. Participants completed the “British” version at the study visit or soon thereafter. Where there was missing data for four or fewer items, a correction factor of 0.25 per missing item was applied, in line with other studies [16].

For categorical analyses, hyposmia was defined as UPSIT score ≤ 33 for males, ≤ 34 for females, and anosmia as UPSIT score ≤ 18 [15]. Normative data for the UPSIT British version have not been published; a comparison to norms for the UPSIT American version is shown in Table 1.

### Neuropsychological testing

The cognitive battery included the Mini-Mental Status Examination (MMSE), Logical Memory, Digit-Symbol Substitution Test, and the Face-Name test [12]. These tests were combined into a modified version of the Preclinical Alzheimer Clinical Composite (PACC) score as described in Lu et al. [17].

### Imaging

Participants underwent PET-MRI scanning on the same 3-T Siemens Biograph mMR scanner [12]. β-amyloid deposition was assessed over a 10-min period, 50 min after injection of 18F-florbetapir (370 mBq). A standardised uptake value ratio (SUVR) was generated from a grey matter cortical composite, with eroded white matter as the reference region. Gaussian mixture models determined a SUVR cut-point of 0.6104 to categorise binary amyloid status.

Hippocampal volume, entorhinal cortex thickness and AD signature cortical thickness were used as markers of neurodegeneration [4, 5]. Hippocampal volumes were determined using STEPS [18] with manual edits where appropriate. AD signature cortical thickness (a composite of temporal cortex regions as described in [19]) and entorhinal cortex measurements were determined using Freesurfer 6.0. Total intracranial volume was calculated using SPM12 (Statistical Parametric Mapping, https://www.fil.ion.ucl.ac.uk/spm/) [20]. White matter hyperintensity volume (WMHV) was derived using Bayesian Model Selection (BaMoS) [21].

### Statistical methods

Data were analysed using Stata 14.1 (StataCorp LP). Chi-squared or Wilcoxon rank-sum tests were used for unadjusted analyses comparing OI category with binary or continuous demographic variables, respectively. Logistic regression was used for adjusted analysis of (binary) amyloid status, linear regression for hippocampal volumes, AD signature cortical thickness, entorhinal cortex thickness and PACC score. As WMHV was non-normally distributed, we used a general linear model with gamma log link. For each of these outcomes, we fitted models with continuous UPSIT score or OI impairment category as the predictor variable, and age, sex, and (where appropriate) TIV as covariates.

## Results

Full data on 389 individuals were available for analysis: mean age at visit was 70.6 (SD 0.68) years, and 50.8% were female. Table 1 compares the distribution of UPSIT scores in this cohort with those of a large cohort of similar age assessed using the UPSIT (American version); the distribution of scores is similar.

Demographic and background features of the normosmic (32.9%), hyposmic (64.5%) and anosmic (2.6%) groups are shown in Table 1. There were no significant differences in sex, age, socio-economic position, smoking, history of head injury, ApoE4 status, or MMSE score between groups.

**Fig. 1:**
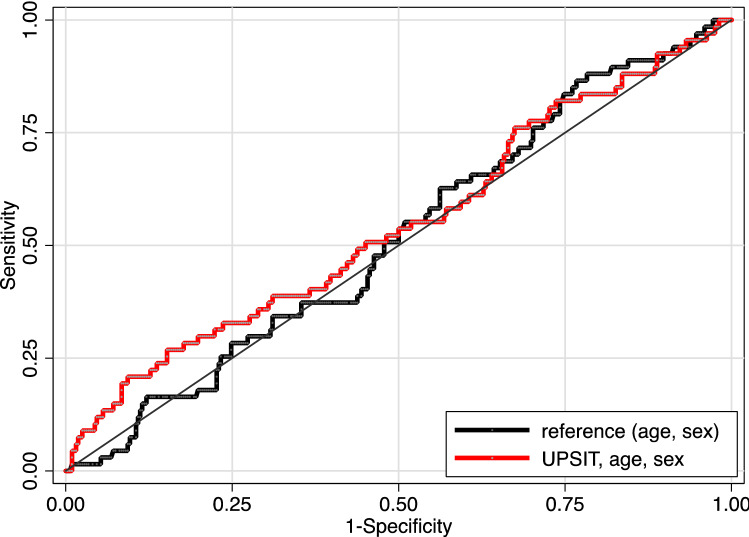
Receiver operating characteristic curve illustrating the predictive value of age, sex and UPSIT score for amyloid status. Area under the curve for age and sex alone, 0.517 (95% confidence interval: 0.444–0.590), versus 0.545 (95% confidence interval 0.465–0.624) when UPSIT score is added to the model *p* = 0.466). This indicates that the addition of UPSIT score has very limited additional discriminatory value to predict amyloid status

There was no significant relationship between continuous UPSIT score and binary amyloid status, adjusting for age and sex (OR 1.04, 95% CI 0.98–1.10, *p* = 0.24). There was no evidence that adding UPSIT score to a base model of age and sex improved prediction of amyloid status (Fig. 1).

Hippocampal volume, entorhinal cortex thickness, cortical thickness, PACC, or WMHV was not associated with continuous UPSIT scores, or when comparing groups categorically (Table 2) after adjusting for age and sex. There was similarly no relationship between UPSIT score and any of the components of the PACC (data not shown).

**Table 2:** Comparison of distribution of UPSIT scores in the current study (UPSIT: British) to previously published normative values (UPSIT: American), by sex [24]

Percentile	UPSIT score			
	Males		Females	
	UPSIT: American, age 70–74 (*n* = 77)	UPSIT: British, age 69–71 (*n* = 191)	UPSIT: American, age 70–74 (*n* = 87)	UPSIT: British, age 69–71 (*n* = 198)
99	39	39	40	38
75	34–35	35	35–36	35
50	29–30	32	32–33	33
25	24	28	27–28	31

## Discussion

In this study of 389 cognitively normal individuals around the age of 70 years, our main findings were (1) ~ 2/3 of individuals fulfil criteria for hyposmia, and (2) that there were no associations between low scores on olfactory identification testing and imaging evidence of β-amyloid pathology, neurodegeneration or cerebrovascular disease, or cognitive performance.

The strongest associations between olfaction and imaging metrics relevant to AD were reported in the Mayo Clinic cohort [4], which also has the highest average age (79 years). The Harvard cohort (mean age 74 years) [5] found a trend level association with amyloid status, and significant associations with imaging markers of neurodegeneration. Noting that our cohort was ~ 10 and 5 years younger than these, respectively, and as older individuals would be expected to have a shorter time to AD onset, this suggests that if OI impairment is not a useful screening tool for asymptomatic pathology, it may however be useful as a proximity marker for the emergence of cognitive impairment. The finding in smaller studies that associations between OI and imaging markers were strengthened by the inclusion of individuals with MCI (who are closer to disease onset) may also indicate this [8, 9]

Limitations of this study include its cross-sectional design and lack of a marker of tau pathology, as there is evidence from pathological [2] and biomarker [7, 22] studies that tau deposition may be more closely linked to olfactory changes. Longer term follow-up of this cohort and the addition of markers of tau pathology will be able to address the latter and the potential proximal relationship of OI to the development of cognitive impairment. Whether or not olfactory loss than can be seen in patients infected with Covid-19 relates to damage to olfactory epithelium or neuronal injury is the subject of ongoing debate, but at the current time, there is no evidence that this is related to Alzheimer pathology [23].

In summary, the high prevalence of OI impairment in populations at this age and lack of relationship between OI and markers of β-amyloid and neurodegeneration we find, indicate that the UPSIT is unlikely to be a reliable predictor of preclinical AD in its very earliest stages (Tables 3, 4).

**Table 3:** Associations between demographic factors and olfactory identification impairment

	Normosmia (*n* = 128)	Hyposmia (*n* = 251)	*p*^a^	Anosmia (*n* = 10)	*p*^a^
Sex, female [*n*, (%)]	62 (48.4)	134 (53.4)	0.362	2 (20.9)	0.082
Age, years [mean, (SD)]	70.7 (0.62)	70.6 (0.70)	0.181	70.6 (0.95)	0.790
SEP, manual occupations [*n*, (%)]	22 (17.2)	34 (13.6)	0.345	2 (20.0)	0.821
Current or former smoking [n, (%)]	80 (62.5)	166 (66.1)	0.483	6 (60.0)	0.875
Head injury prior to age 69–71 [*n*, (%)]	22 (17.2)	28 (11.2)	0.101	2 (20.0)	0.821
ApoE 4 carrier [*n*, (%)]	39 (29.7)	72 (28.9)^b^	0.472	4 (40)	0.495
MMSE score [median, (IQR)]	30 (29–30)	30 (29–30)	0.973	29.5 (29–30)	0.565

*SEP* socio-economic position, *MMSE* mini-mental status examination

^a^*p* value compared to normosmia, determined by chi-square test (categorical variables) or Wilcoxon rank-sum test (continuous variables)

^b^*n* = 249 for this variable

**Table 4:** Associations between continuous and categorical UPSIT scores and imaging outcomes in 389 cognitively normal individuals at age 69–71

	Continuous analyses			Categorical analyses					
	UPSIT score			Hyposmia			Anosmia		
	*β*	95% CI	*p*	*β*	95% CI	*p*	*β*	95% CI	*p*
*Associations by linear regression (β* *coefficient)*									
Hippocampal volume (mL)^b,c^	− 0.002	− 0.004, 0.011	0.805	− 0.075	− 0.200, 0.050	0.239	− 0.162	− 0.540, 0.217	0.401
Entorhinal cortex thickness (mm)^a,c^	0.001	− 0.004, 0.006	0.671	0.010	− 0.039, 0.059	0.693	− 0.041	− 0.191, 0.108	0.587
Cortical thickness (mm)^a^	0.001	− 0.001, 0.003	0.156	− 0.003	− 0.022, 0.017	0.771	0.001	− 0.058, 0.059	0.986
Global cognitive score (modified PACC)^a^	− 0.003	− 0.017, 0.011	0.657	0.001	− 0.141, 0.140	0.992	0.252	− 0.172, 0.677	0.244
*Associations by generalised linear model (exponentiated* *β* *coefficient)*									
White matter hyperintensity volume (mL)^b,d^	0.992^e^	0.967, 1.016	0.609	1.153^e^	0.906, 1.468	0.247	1.280^e^	0.624, 2.628	0.501

^a^Adjusted for age and sex

^b^Adjusted for age, sex and total intracranial volume

^c^Expressed as the mean of right and left

^d^*n* = 377 for this outcome

^e^Expressed as exponentiated *β* coefficient; value represents the ratio change in WMHV per 1 point increase in UPSIT score, or between groups

## Electronic supplementary material

Below is the link to the electronic supplementary material.

Supplementary file1 (DOCX 25 kb)

## Data Availability

A data-sharing policy is available on the NSHD Data Sharing website https://www.nshd.mrc.ac.uk/data.
