# Supplementary material for: Olfactory testing does not predict β-amyloid, MRI measures of neurodegeneration or vascular pathology in the British 1946 birth cohort
Source: J Neurol. 2020 Jun 24;267(11):3329–36. doi: 10.1007/s00415-020-10004-4 (PMC7311798; doi:10.1007/s00415-020-10004-4)
Supplement: Supplementary file 1 — Supplementary file1 (DOCX 25 kb) [file 415_2020_10004_MOESM1_ESM.docx]

**Olfactory testing does not predict ß-amyloid, MRI measures of neurodegeneration or vascular pathology in the British 1946 birth cohort.**

**Supplementary data:**

Attended Insight 46 study visit: n=502

**Exclusions**

**Exclusions – participant characteristics:**

Neurodegenerative Diseases: n=7

Mild cognitive impairment: n= 8

Nasal disorders: n=34

**Exclusions -incomplete data:**

Did not complete scanning protocol/failed imaging quality control: n=36

UPSIT not completed: n=24

UPSIT results invalid n=4

**Neuroimaging analyses:**

Hippocampal volume, cortical thickness analyses: n=389

Failed white matter quality control: n=12, so n=377 included in these analyses

**Demographic, cognitive** analyses: n=389

**Final sample:**

n=389

Supplementary Figure 1: Participant exclusions, flow chart
